# Comparison of Dose Response Models for Predicting Normal Tissue Complications from Cancer Radiotherapy: Application in Rat Spinal Cord

**DOI:** 10.3390/cancers3022421

**Published:** 2011-05-18

**Authors:** Magdalena Adamus-Górka, Panayiotis Mavroidis, Bengt K. Lind, Anders Brahme

**Affiliations:** Department of Medical Radiation Physics, Karolinska Institutet and Stockholm University, Stockholm S-17176, Sweden; E-Mails: magdalena.adamusgorka@gmail.com (M.A.-G.); bengt.lind@ki.se (B.K.L.); anders.brahme@ki.se (A.B.)

**Keywords:** radiobiological models, NTCP, spinal cord complications

## Abstract

Seven different radiobiological dose-response models have been compared with regard to their ability to describe experimental data. The first four models, namely the critical volume, the relative seriality, the inverse tumor and the critical element models are mainly based on cell survival biology. The other three models: the Lyman (Gaussian distribution), the parallel architecture and the Weibull distribution models are semi-empirical and rather based on statistical distributions. The maximum likelihood estimation was used to fit the models to experimental data and the χ^2^-distribution, AIC criterion and F-test were applied to compare the goodness-of-fit of the models. The comparison was performed using experimental data for rat spinal cord injury. Both the shape of the dose-response curve and the ability of handling the volume dependence were separately compared for each model. All the models were found to be acceptable in describing the present experimental dataset (p > 0.05). For the white matter necrosis dataset, the Weibull and Lyman models were clearly superior to the other models, whereas for the vascular damage case, the Relative Seriality model seems to have the best performance although the Critical volume, Inverse tumor, Critical element and Parallel architecture models gave similar results. Although the differences between many of the investigated models are rather small, they still may be of importance in indicating the advantages and limitations of each particular model. It appears that most of the models have favorable properties for describing dose-response data, which indicates that they may be suitable to be used in biologically optimized intensity modulated radiation therapy planning, provided a proper estimation of their radiobiological parameters had been performed for every tissue and clinical endpoint.

## 1. Introduction

The study of the dose-response relations in radiation therapy is important for improving quantification and knowledge about the mechanisms influencing the response of organs and tissues to radiation therapy. It is important to know the expected response level in normal tissues when irradiating a patient, since the aim of radiation therapy is to eradicate the tumor while sparing healthy normal tissues as far as possible. This is particularly important when using radiobiologically optimized radiation therapy where both the therapeutic effect and adverse normal tissue damage need to be accurately quantified in order to maximize the treatment outcome. Most often, the dose to the tumor is limited by the tolerance of the surrounding normal tissues. It is essential to understand the underlying biological processes for selecting the proper model, which can more accurately describe the normal tissue response and determine tissue tolerance in different situations. In this way, it will be possible to estimate the quality of life after the treatment by calculating the probability of tumor cure or local control and the associated risk of treatment related morbidity.

There exist several types of volume effects, defined by the decrease in tissue function or increase in the probability of having a specific endpoint with increasing irradiated volume. The response of a tissue to radiation depends on the organization of its sensitive functional subunits, the volume of the irradiated tissue and possible irradiation of associated organs, and finally on the ability of the different cell types to maintain the tissue or organ function. The latter is often dependent on the way different types of cells are organized into functional subunits (FSUs) [1,2]. The functional arrangement of the FSUs of a tissue is critical for its radiation response both with regard to the dose distribution and the volume irradiated. The FSUs can be functionally arranged in series, parallel or have a mixed serial-parallel or cross-linked organization. Serially arranged FSUs are organized like links of a chain, such as the insulating myelin cells of the axons in the spinal cord. In such a case, the function of each FSU is critical for the function of the organ and elimination of any one of them may result in a measurable loss of function or an increased probability for complications. Therefore, in serial tissues the maximum dose largely determines the therapeutic response. However, when the FSUs are functionally organized in parallel, there is a redundancy where neighboring FSU can take over the function. In such tissues, the volume dependence of the dose-response relation is very significant since the response in a small high dose volume can be almost fully compensated by the surrounding FSUs. In parallel tissues, the mean dose is therefore the most important factor determining the clinical effect. Mixed serial-parallel tissue organizations are the most common and general way of tissue organization, combining the structure and function of the two basic arrangements [2]. The relative seriality model is designed to describe the gradual change in response from a closely serial tissue to one which is largely parallel.

Spinal cord is a critical normal tissue that almost at all cost should be spared during radiation therapy. It is an example of an organ with high serial arrangement of its functional subunits. It is built of nerve cells—Neurons, the axons of which are arranged in bundles along the organ. The characteristic H-shaped pattern on the spinal column cross section is a result of the arrangement of the nerve cell bodies and axons within the cord. The inner part, creating the H-letter shape consists of gray matter, while the white matter creates a more lipid rich, pale surrounding.

It is well known that the material building the gray matter is mainly nerve cell bodies, while axons and the associated myelin cells are the ones constituting the white matter. In mammalian nerve tissue, the axons (e.g., motor neurons or sensory neurons) are equipped with a special layer of insulation, namely the myelin sheath. The myelin sheath is created by Schwann cells surrounding the axons of a neuron, increasing the integrity, speed and information content of the transmitted signal.

There are often very serious consequences for exceeding the tolerance dose of normal tissues. As far as the latency period is concerned, radiation response can be divided into an early and a late occurring damage. There is a close correlation between the time of appearance of radiation-induced damage and the normal proliferative activity for a given tissue. The higher the rate of normal cell turnover, the faster the onset of the damage. In slowly proliferating tissues, such as spinal cord, the induction of radiation damage is considerably delayed in time. The late types of radiation-induced damage, in case of spinal cord myelopathy and paralysis, consist of two main endpoints: White matter necrosis and demyelination, occurring usually between six to eighteen months after irradiation, followed by vascular damage with an onset that ranges between one to four years.

Modeling of normal tissue response to radiation has become an important domain of modern radiation therapy. Numerous models have been developed during the years to help in determining the optimal treatment. The process of creating such models usually involves many simplifying assumptions. The damage induction is considered stochastic, whereas the survival of cells follows either binomial or poisson statistics. The organ response is assumed to depend on either the response of individual cells and/or the response of the FSU. All the cells as well as all the FSUs are assumed to respond identically. The isoeffect relationships do not depend on the level of response and equal dose fractions are assumed to cause equal effects, provided the time separation is sufficient. Two connected levels of radiation response are generally modeled, namely survival of cells and response of an organ. Many models originate from an expression that describes cell survival and they incorporate this expression in the formula that describes the relation between dose and organ function. However, other models are purely phenomenological, which means that there is not any explicit formula for cell survival included. A radiobiological model, to be considered reliable, has to fulfill certain requirements. It should appropriately predict the shape of the dose-response curve as well as it should duly handle the volume and fractionation effects.

Numerical quantitative comparisons of existing dose-response models have been done by many authors [3-9]. However, despite the great interest in this subject an important issue has not been taken into account in these studies, namely the separation of the volume effect from the dose-response of the whole organ. Being able to separate these two different phenomena should not only allow estimation of the accuracy and clinical validity of the models, but also make it possible to investigate the diversity of the models and emphasize the differences between them. A model combining accurate dose-response description together with precise volume effect handling is required for accurate optimization of the treatment outcome.

## 2. Materials and Methods

**Figure 1.**
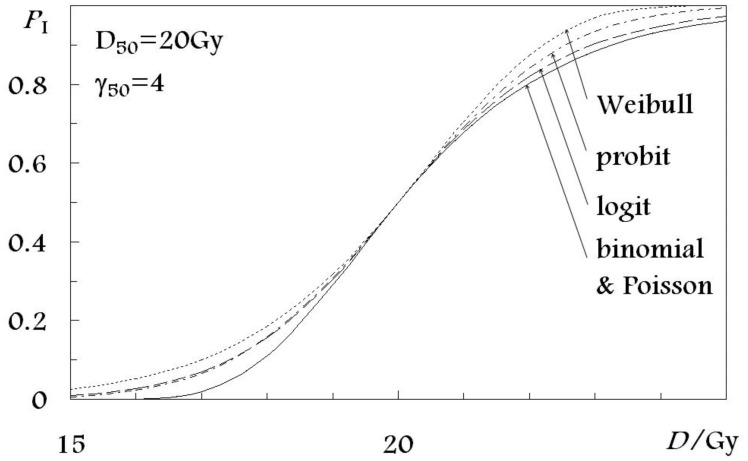
Statistical distributions used in NTCP models to describe the shape of the dose-response curve.

Dose-response models can be categorized into several groups based on the statistical distribution they use for describing the sigmoid shape of the dose-response curve (Figure 1). The five distributions used in the models investigated in the present study are: the poisson, binomial, probit, logit and Weibull distributions. They constitute a basis for the following seven radiobiological models for normal tissue complication probability: (a) the Critical volume model [10] based on the binomial distribution for the dose-response curve shape; (b) the Relative Seriality model [2]; (c) the Inverse tumor model [2]; and (d) the Critical element model [11], all of which are based on the Poisson statistics; (e) the Lymna model [12] based on the Gaussian distribution or the probit function; (f) the Parallel architecture model [13] using the logit expression; and (g) the Weibull distribution model [14] based on the Weibull distribution. The first four models are using cell-survival-based response (Poisson and binomial distribution for the shape of the dose-response curve), while the other three models are more phenomenological. To rationalize the comparison in this paper, the expressions for NTCP of all the models have been rewritten in terms of *D*_50_, which is the dose that is associated with the 50% response probability and γ_50_, which is the gradient of the dose-response curve at the level of the 50% response probability (Table 1).

### 2.1. Distributions Used for the Shape of Dose-Response Curve

#### 2.1.1. The binomial distribution

Assuming *N_0_* functional subunits and a probability of FSU survival, *S(D)*, at a dose *D* gives the following probability of response:

(1) $$\text{P}\left(\text{D}\right)={\left(1-\text{S}\left(\text{D}\right)\right)}^{{\text{N}}_{0}}$$

The following simple equation for exponential cell survival is used [15]:

(2) $$\text{S}\left(\text{D}\right)={\text{e}}^{-nd/D_{0}}$$

where *D = n·d* is the total dose, *d* is the dose per fraction, *n* is the number of fractions and *D*_0_ is the dose giving on average one lethal hit per FSU. Together with the binomial model for response, equation (1), the dose giving 50% response probability, *D*_50_, and the maximum value of the normalized dose-response gradient, γ̃ [16], become:

(3) $$D_{50}=-D_{0}\ln\left(1-\frac{1}{2^{1/N_{0}}}\right)$$

and

(4) $$\tilde{\gamma }=\ln N_{0}{\left(1-\frac{1}{N_{0}}\right)}^{N_{0}-1}$$

Respectively. γ̃ is fundamentally defined by:

(5) γ˜≡D˜·P'(D˜),whereP'(D˜)=maxD(∂P(D)∂D)

For large *N*_0_ the expressions for *D*_50_ and γ̃; for the binomial model become identical to the expressions for the Poisson model (Figure 1).

*The critical volume model* has been developed by Niemierko and Goitein [17]. The probability *P* that more than *M* of the *N* FSUs are killed is given by the cumulative binomial probability:

(6) $$P=\sum_{t=M+1}^{N}P_{t}=\sum_{t=M+1}^{N}\left(\begin{array}{l} N\\ t \end{array}\right)P_{\text{FSU}}^{\text{t}}{\left(1-P_{\text{FSU}}\right)}^{N-t}$$

where *P_t_* is the probability that *t* of the *N* FSUs are killed, $\left(\begin{array}{l} \text{N}\\ \text{t} \end{array}\right)$ is the binomial coefficient, ${\text{P}}_{\text{FSU}}^{\text{t}}$ is the complication probability for *t* functional subunits, while *P*_FSU_ is the complication probability for one FSU. The NTCP for the entire inhomogeneously irradiated organ can be calculated using equation (6) with the *P_FSU_* being replaced by the effective complication probability for one FSU:

(7) $${\text{P}}_{\text{FSU}}^{\text{eff}}=\frac{1}{{\text{N}}_{\text{P}}}\sum_{\text{i}=1}^{{\text{N}}_{\text{P}}}{\text{P}}_{\text{FSU}}^{\text{i}}\left({\text{D}}_{\text{i}}\right)$$

where *N*_p_ is the number of calculation points inside the organ of interest, $P_{\text{FSU}}^{i}$ is the complication probability for the *i*^th^ FSU and *D_i_* is the corresponding dose received. Due to the difficulties in calculating the cumulative binomial distribution a normal distribution approximation suitable for numerical calculations is often used.

(8) $$\text{P}=\sum_{\text{t}=\text{M}+1}^{\text{N}}\left(\begin{array}{l} \text{N}\\ \text{t} \end{array}\right){\text{P}}_{\text{FSU}}^{\text{t}}{\left(1-{\text{P}}_{\text{FSU}}\right)}^{\text{N}-\text{t}}\approx \frac{1}{\sigma_{\text{FSU}}\sqrt{2\pi }}\int_{-\infty }^{\text{M}}\exp\left(-\frac{{\left(\text{x}-\text{N}\cdot {\text{P}}_{\text{FSU}}\right)}^{2}}{2\sigma_{\text{FSU}}^{2}}\right)\text{dx}$$

where:

(9) $$\sigma_{\text{FSU}}=\sqrt{\text{N}\cdot {\text{P}}_{\text{FSU}}\left(1-{\text{P}}_{\text{FSU}}\right)}$$

Such an approximation is more accurate for large *N·P_FSU_*(*1−P_FSU_*) values.

#### 2.1.2. The Poisson distribution

The Poisson distribution is the limiting case of the binomial distribution when *N*_0_ is large and presents the probability of complications in normal tissue by:

(10) $$\text{P}\left(\text{D}\right)={\text{e}}^{-{\text{N}}_{0}\cdot \text{S}\left(\text{D}\right)}$$

where *N*_0_ is the number of functional subunits [1] and *S*(*D*) is a probability of an FSU surviving a dose, *D*. Thus, *N*_0_*·S*(*D*) becomes the average number of FSUs surviving a dose, *D*. Using the exponential cell survival equation (2) for clonogenic cell survival, together with the Poisson model, equation (10), for response, *D*_50_, and γ̃, become:

(11) $${\text{D}}_{50}={\text{D}}_{0}\left(\ln{\text{N}}_{0}-\ln\ln2\right)$$

and

(12) $$\tilde{\gamma }=\frac{\ln{\text{N}}_{0}}{\text{e}}$$

Respectively, where e is the base of the natural logarithm. For models based on Poisson statistics the maximum slope of the dose-response relation is at the dose giving 37% probability of response. For that reason γ̃ is sometimes denoted as γ_37_. In order to facilitate the comparison between different models, also those using γ_50_, the following transformation can be used:

(13) $$\gamma_{50}=\frac{\ln2}{2}\left(\text{e}\tilde{\gamma }-\ln\ln2\right)$$

*The relative seriality model* was developed to better account for the functional organization of FSU's [2]. An arbitrary combination of serial and parallel organized FSUs can be considered. For this model, normal tissue complication probability is mathematically expressed by:

(14) $$\text{P}\left(\text{D},\text{V}\right)={\left[1-{\left(\text{P}{\left(\text{D}\right)}^{\text{s}}\right)}^{\text{V}/{\text{V}}_{\text{ref}}}\right]}^{1/\text{s}}$$

where *V/V_ref_* is the volume fraction being irradiated to dose *D, s* is the parameter which expresses the degree of seriality (the value varies from *s* close to zero for nearly parallel organs and upwards for increasing seriality), *P*(*D*) is given, e.g. by the Binomial or Poisson expression (10).

*The inverse tumor model* [2] was based on a simplistic inverse tumor response and fundamentally it is not a real normal tissue model. The NTCP may then be approximated in the following way:

(15) $$\text{P}\left(\text{D},\text{V}\right)=\exp\left[-{\text{N}}_{0}{\text{e}}^{-\left(\text{D}/{\text{D}}_{0}\right)}+\text{k}\ln\left(\text{V}/{\text{V}}_{\text{ref}}\right)\right]$$

where the free parameter *k* takes into account the importance of the volume effect in the tissue.

*The critical element model* [17] is a simplified case of the relative seriality model, obtained by putting *s* = 1 into equation (14). The expression for NTCP is then given by:

(16) $$\text{P}\left(\text{D},\text{V}\right)=1-{\left(1-\text{P}\left(\text{D}\right)\right)}^{\text{V}/{\text{V}}_{\text{ref}}}$$

where *P*(*D*) is given by equation (10).

#### 2.1.3. The Normal distribution

Using the normal distribution function (the probit function) for the response results in the following expression:

(17) $$\text{P}\left(\text{D}\right)=\frac{1}{2}\left(1-\text{Erf}\left[\gamma_{50}\sqrt{\pi }\left(1-\frac{\text{D}}{{\text{D}}_{50}}\right)\right]\right)$$

*The Lyman (Gaussian) model* was developed by Lyman [12] based on the error or probit function form. In this case, the normal tissue complication probability is given by the following expression:

(18) $$\text{P}\left(\text{D},\text{V}\right)=\frac{1}{\sqrt{2\pi }}\int_{-\infty }^{\text{t}}\exp\left(-{\text{t}}^{2}/2\right)\text{dt}$$

where the upper limit, *t* of the normal distribution probability function is defined as follows:

(19) $$\text{t}\left(\text{D},\text{V}\right)=\frac{\text{D}-{\text{D}}_{50}\left(\text{V}/{\text{V}}_{\text{ref}}\right)}{{\text{mD}}_{50}\left(\text{V}/{\text{V}}_{\text{ref}}\right)}$$

and

(20) $${\text{D}}_{50}\left(\text{V}/{\text{V}}_{\text{ref}}\right)={\text{D}}_{50}\left(1\right){\left(\frac{\text{V}}{{\text{V}}_{\text{ref}}}\right)}^{-\text{n}}$$

The model contains four free parameters: *D_50_, n, m* and *V*_ref_. *D_50_* and *V*_ref_ were defined above, while *D*_50_(1) is the tolerance dose for 50 % complications for uniform whole organ irradiation, *D*_50_(*V/V*_ref_) is the 50 % tolerance dose for uniform partial organ irradiation. The volume dependence of the complication probability is determined by the parameter *n*, which quantifies the sensitivity of *P* to the irradiated volume of the organ. The slope of the dose response curve is governed by the value of theparameter *m*. The slope parameter *m* is inversely proportional to *γ*_50_ through the relation $\text{m}=\frac{1}{\gamma_{50}\sqrt{\pi }}$.

#### 2.1.4. The Logit distribution

The logit distribution is an analytical sigmoidal shaped curve commonly used in biology defined in the following way:

(21) $$\text{P}\left(\text{D}\right)=\frac{1}{1+{\left(\frac{{\text{D}}_{50}^{\text{FSU}}}{\text{D}}\right)}^{\text{k}}}$$

*The parallel architecture model* [11,13,18] presents NTCP as an increasing function of the number of FSUs inactivated by radiation. The probability *p* that a dose *D* inactivates an FSU is given by the logit expression (21) where the slope parameter *K =* 4·*γ*_50_. The above sigmoid dose-response function, *P*(*D*) is assumed to describe the probability of damaging a subunit at a given biologically equivalent dose. Apart from the assumption that biologically equivalent doses can be calculated from the linear-quadratic formula, no connections of this probability with any underlying vascular mechanism of radiation injury or identification of the subunits involved has been attempted. Instead it has been chosen to describe the subunit response phenomenologically, using a logistic function of dose parameterized in terms of the dose *D_50_* at which 50% of the subunits are damaged, and the slope parameter *k*, that determines the rate at which the probability of damaging a subunit increases with dose. For a given dose matrix the total fraction of FSUs, being inactivated is given by the sum over all the individual contributions:

(22) $$\text{f}=\sum {\text{v}}_{\text{i}}\text{P}\left({\text{D}}_{i}\right)$$

where *D_i_* and *v_i_* are the dose and the volume fraction of the *i^th^* voxel and *f* is the fractional damage. To fit the parallel architecture model to clinical data, expressions for both *P*(*D*) and the statistical distribution of functional reserves over the patient population are required. Normal tissue complication probability for a given Dose Volume Histogram (DVH) is calculated from the equation:

(23) $$P\left(D,V\right)=\frac{1}{\sqrt{2\pi \sigma^{2}}}\int_{0}^{\text{f}}\exp\left[-\frac{{\left(v-v_{50}\right)}^{2}}{2\sigma^{2}}\right]\mathrm{dv}$$

In which it is assumed that the cumulative functional reserve distribution can be described as a displaced error function and quantified by the mean value of the functional reserve, *v*_50_ and the width of the functional reserve distribution, *σ*. In this equation, *v* is the partial organ volume being irradiated [13,19,20].

#### 2.1.5. The Weibull distribution model

In this model the mathematical expression for NTCP, *P_I_* is based on a modified Weibull function [21]:

(24) $$\text{P}\left(\text{D},\text{V}\right)=1-\exp\left[-\frac{{\left(\text{D}{\left(\frac{\text{V}}{{\text{V}}_{\text{ref}}}\right)}^{\text{b}}\right)}^{{\text{A}}_{2}}}{{\text{A}}_{1}}\right]$$

where *A*_1_, *b and A_2_* are the three model parameters, which are determined from clinical data [14]. This can be rewritten in terms of *D_50_* and γ_50_ as:

(25) $$\text{P}\left(\text{D}\right)=1-\exp\left[-\ln2{\left(\frac{\text{D}}{{\text{D}}_{50}}\right)}^{\frac{2}{\ln2}\gamma_{50}}\right]$$

### 2.2. Statistical Methods

The model inter-comparison was performed both with and without considering the volume effect. This was made in order to separately evaluate and compare: (a) the accuracy by which the different models fit the shape of the dose-response curves from uniform dose irradiation; and (b) the ability of the different models to account for the volume effect. To be able to judge each of the above phenomena individually, we tried to separate them by removing the volume effect. This was achieved through a separate fit of the models for each spinal cord length, which was done by making a fit to each of the irradiated length of spinal cord separately without taking the volume dependence of the model into consideration, assuming that each partially irradiated length is a separate unit. Each of the models had a total of six free parameters to be estimated due to the cancelling of the ones describing the volume dependence.

The Lyman (Gaussian) model is the most widely used model in the literature. For this reason the authors chose to use this model as a reference in order to be able to project the findings of the present study to other clinical studies where the Lyman model has been used for analyzing the treatment outcome data.

To perform the fitting of the models to experimental data, the maximum likelihood method was used [21]. The maximum likelihood method is perhaps the most powerful estimation for two particular types of problems: (i) low-statistics experiments with insufficient data to satisfy the requirement of Gaussian statistics for individual histogram bins; and (ii) experiments in which the fitting function corresponds to a different probability density function for each measured event (meaning that each patient has a different weight in the calculations which is determined by the different dose distribution received). This method estimates the values of the model parameters that are more likely to produce a pattern of responses similar to the one of the observed clinical data. The larger the value of the likelihood function, the larger is the weight of evidence in favor of a given set of parameters. The logarithm of the likelihood function is often used for computational convenience. The logarithm of the likelihood function is the logarithm of probability that the experiment ends in the way it actually did. The larger the Log-likelihood value, the better the respective fit. This method was used in our study in order to both compare the overall fitting of all the investigated models to the experimental data *(i.e.*, including the volume effect) as well as to compare the fitting of the models without accounting for the volume effect.

After fitting the models to the clinical data, the goodness of fit of the models and their parameters was evaluated by the χ^2^-test, which was applied as suggested by Baltas and Grassman [4]. The χ^2^ value, although referred to as a measure of goodness of fit, actually represents a measure of lack of fit and it should thus be as low as possible. This means that the smaller the χ^2^ or the reduced χ^2^ values (taking into account the number of degrees of freedom, DF that is the number of data points in the particular dataset reduced by the number of parameters in the respective model), the better the overall fit of the model or the better the dose-response curve agrees with the experimental data, when the volume effect has been removed.

An inter-comparison of the fitting of the models to the experimental data was done using the F-test method. The main principle of this method is to perform a comparison between a given model and the Lyman model with volume effect, which is the reference model, by comparing their fitted results to the experimental data. The F-test value is a probability distribution calculated for the χ^2^ value of the reference model divided by the χ^2^ value for the model under investigation. The smaller the F-test value, the better the compared model is in comparison to the reference model. The value *of p* = 0.5 means that the compared model and the reference one are identical; for *p* < 0.5 the compared model is better than the reference one, while for *p* > 0.5 the reference model is better. For the inter-comparison of the overall fits of the models, the commonly used Gaussian distribution model was chosen as reference. In order to compare the fits of the models without accounting for the volume effect, their overall fits were compared with the corresponding fits without considering the volume effect for each individual model separately. For a more thorough discussion of the mentioned methods [22].

This analysis was based on a goodness-of-fit evaluation. Under the assumption of Gaussian errors around the true function describing survival, the model behavior was studied at different dose ranges for each clinical endpoint. The chi-square values were calculated and the corresponding *p*-values were determined taking into account the degrees of freedom.

Also, the Akaike's information criterion [23] was used to compare the accuracy and complexity of the different models. In the general case, the AIC is mathematically expressed as follows:

(26) $$\text{AIC}=\chi^{2}+2\text{k}$$

where χ^2^ is the chi-square value for the estimated model and *k* is the number of parameters in the statistical model. A lower Akaike number for a model means superiority of that model.

### 2.3. Experimental Data

The models were fitted to experimental data for paralysis after irradiation of spinal cord of rats [24], as presented in Table 2. The doses with which the corresponding spinal cord lengths were irradiated as well as the number of responders in the different irradiated groups are given. Using this data, the different parameters of the models were calculated for each of the endpoints using all the irradiated spinal cord lengths. The results were used to make an inter-comparison between the predictions of the different models.

## 3. Results

### 3.1. Comparison of the Models Using the Maximum Likelihood Function

**Figure 2.**
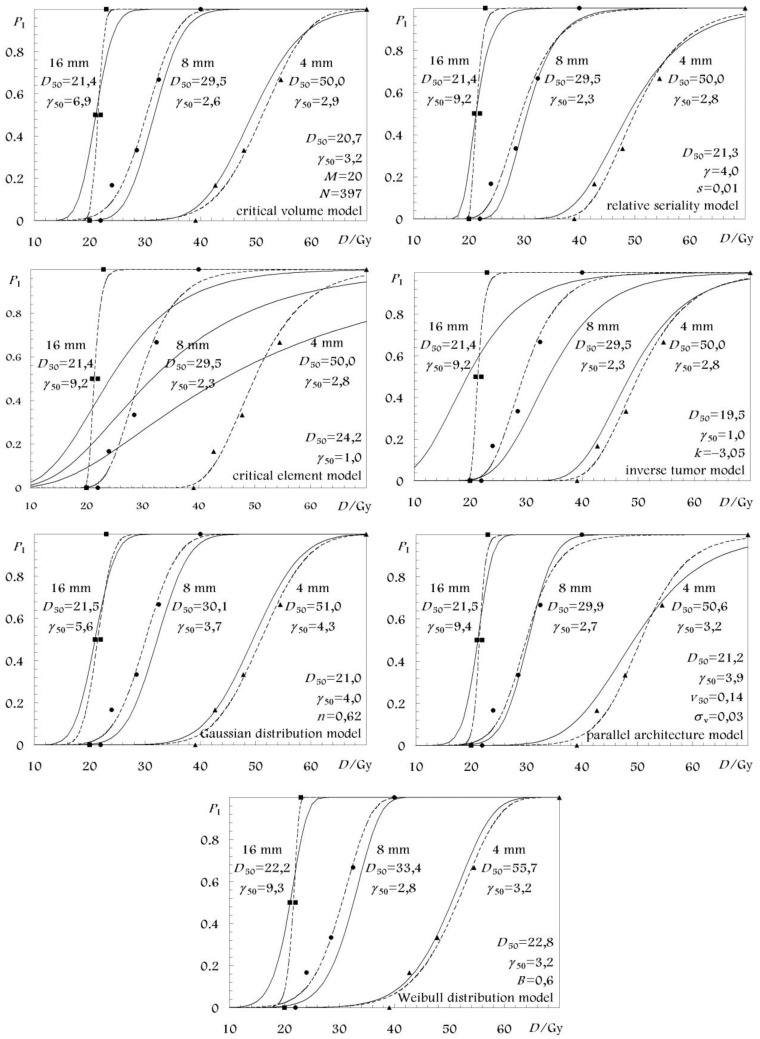
Volume and dose-response curves for white matter necrosis of different lengths of rat cervical spinal cord. The solid lines give the combined best fitting. The dashed lines have been fitted to each of the irradiated spinal cord segment lengths separately, *i.e.*, without any volume effect.

**Figure 3.**
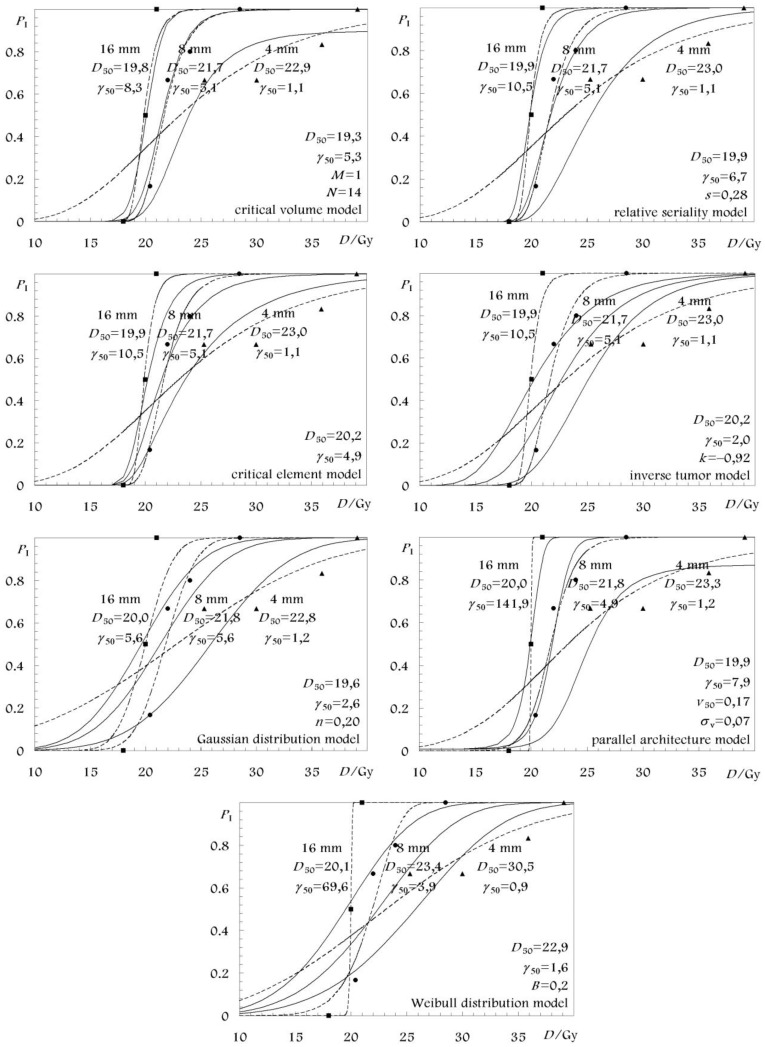
Volume and dose-response curves for vascular damage of different lengths of rat cervical spinal cord. The solid lines give the combined best fitting. The dashed lines have been fitted to each of the irradiated spinal cord segment lengths separately, *i.e.*, without any volume effect.

The best estimates as well as the 68% confidence intervals of the parameters of all the models are given in Table 3 for the white matter necrosis and in Table 4 for the vascular damage endpoint. In these tables only the parameter values from the fittings that considered volume effect are presented for each model. The respective parameter values from the fittings of the separate spinal cord segment lengths are shown in Figure 2 and Figure 3 where the associated dose-response curves are plotted together with those which are based on the parameter values from Table 3 and Table 4. For all the models, the reference volume, *V*_ref_ was defined by the reference spinal cord length of *L*_0_ = 16 mm corresponding to a relative volume *v* = 1 [4,25].

These values were determined using the maximum likelihood method to fit the complete set of experimental data (with volume effect) and the different spinal cord segment lengths, separately (without volume effect) in each case (white matter necrosis, vascular damage). For white matter necrosis, the degrees-of-freedom (DF) is 14 − *k* when volume effect is considered whereas it is 14 − 3 * 2 = 8 (three spinal cord segments with two parameters to be determined for each segment) when volume effect is not considered. Similarly, for the vascular damage, the corresponding DF is 11 − *k* and 11 − 3 * 2 = 5.

For the white matter necrosis, as expected, the maximum value of the Log-likelihood function is higher in the fittings without volume effect since more parameters are used to fit each spinal cord segment length separately compared to the fittings with volume effect in the models. However, among the different models, the higher values were received by the Parallel architecture (−34.3) and the Relative Seriality (−35.3) models with volume effect and the Weibull (−31.6) and the Relative Seriality (−31.9) models without volume effect. However, the maximum value of the Log-likelihood function is only an approximate descriptor of the goodness-of-fit and it does not account for the mean and variance of the Log-likelihood function distribution around its maximum.

Similar results hold for the vascular damage. Among the different models, the higher values of the maximum of the log-likelihood function were received by the Relative Seriality (−26.1) and the Critical volume (−26.2) models with volume effect and the Weibull (−24.7) and the Parallel architecture (−24.7) models without volume effect.

### 3.2. Model Inter-Comparison Using the χ^2^ Distribution, AIC and F-Test Measures

For the white matter necrosis, based on the χ^2^ and *P*_χ_ (probability of the χ^2^ distribution), the best fittings are achieved by the Weibull (10.33, 0.50) and the Lyman (11.46, 0.41) models with volume effect and the Critical volume (4.57, 0.80) and the Weibull (4.26, 0.83) models without volume effect. However, the values of χ^2^ and *P*_χ_ do not precisely account for the number of model parameters that have to be determined. More accurate descriptors for the goodness-of-fit and model inter-comparison are the AIC and the F-test measures. By using the AIC measure, the best fits are achieved by the **Weibull (16.33)** and the Lyman (17.46) models with volume effect and the **Weibull (16.26)** and the Critical volume (16.57) models without volume effect. Finally, when the F-test is used, the best fits are achieved by the **Weibull (0.43)** and the Lyman (0.50) models with volume effect and the **Lyman (0.84)** and the Parallel architecture (0.87) models without volume effect.

For the vascular damage, based on the χ^2^ and *P*_χ_ the best fits are achieved by the Critical volume (4.11, 0.77) and the Relative Seriality (5.03, 0.75) models with volume effect and the Relative Seriality (1.51, 0.91) and the Parallel architecture (1.80, 0.88) models without volume effect. By using the AIC measure, the best fits are achieved by the **Critical element (9.62)** and the Relative Seriality (11.03) models with volume effect and the **Relative Seriality, Inverse tumor and Critical element (13.51)** models without volume effect. Finally, when the F-test is used, the best fits are achieved by the **Critical volume (0.08)** and the Relative Seriality (0.11) models with volume effect and the **Lyman and Parallel architecture (0.88)** models without volume effect.

The qualitative part of this information can be observed in Figure 2 and Figure 3, where the dose-response curves of the different models based on the different fits are presented. The schematical illustration of the model dose-response curves against the experimental data can been a good indication about the accuracy of the fit.

## 4. Conclusions

Currently, in most clinical practices, when evaluating the fitness of a plan, the mean and maximum or minimum doses, isodose distributions and DVH are typically examined. However, these data do not take into account the biological characteristics of the examined tissue. That is because different treatment plans may deliver different dose distributions to a given normal tissue getting the same response rate. Just as the dose volume histogram chart is a good illustration of the volumetric dose distribution delivered to the patient, so is the dose-response plot as a measure of the expected clinical outcome.

It has been reported that radiobiological evaluation is more sensitive to small changes in dose distribution and the differences observed in the dose-response diagrams comparing different treatment plans are not always reflected in the DVH plots. This is because the way a certain dose distribution affects an organ depends on its radiobiological characteristics. Using the dose-response diagrams together with the dosimetric diagrams, a more complete picture of the effectiveness of a given treatment plan may be given. Consequently, there is an obvious need for radiobiological models that are able to describe quantitatively the normal tissue response and its dependence on the irradiated volume and dose level. In the present study, a comparison of available models has been made. The results of the fit of these models to the experimental data describing rat paralysis caused by white matter necrosis or vascular damage after irradiation gave a good indication that these models show a suitable behavior in describing relevant experimental data.

The results and conclusions of this study are strongly dependent on the accuracy of the radiobiological models and the parameters describing the dose-response relation of the different tissues. However, it is known that all the existing models are based on certain assumptions or take into account certain only biological mechanisms. Furthermore, in clinical practice the determination of the model parameters expressing the effective radiosensitivity of the tissues is subject to uncertainties imposed by the inaccuracies in the patient setup during radiotherapy, lack of knowledge of the inter-patient and intra-patient radiosensitivity and inconsistencies in treatment methodology. Consequently, the determined model parameters and the corresponding dose-response curves are characterized by confidence intervals. So, the expected response of a tissue is known with some uncertainty, which has become clinically acceptable for many cancer sites.

Until now, in clinical practice the different tissues are generally assumed to have homogeneous radiosensitivity. DVHs are a good illustration of the volumetric dose distribution delivered to an organ but spatial dosimetric information gets lost. However, there is increasing evidence that the spatial information of the dose distribution is important in determining treatment complications. Therefore, although DVH simplifies a 3D dose distribution into a 2D plot, such a plot may not be representative of the efficacy of the given treatment technique and therefore may have not a close relation with treatment outcome. The presented models cannot account for any spatially distributed radiosensitivity variation in their present forms and they have to be further developed in order to incorporate such information.

In order to compare the fit of the models to the experimental data four methods were used (the maximum likelihood method, the χ^2^ distribution, the AIC method and the F-test). The results for all the examined models based on the above statistical methods are shown in Table 3 and Table 4.

From the presented results one can clearly see that the range of differences between the different models in fitting the experimental data is large in the case that volume effect is accounted for (F-test: 0.43–0.90 for white matter necrosis and 0.08–0.53 for vascular damage) whereas it is small in the case that the different spinal cord segment lengths are fitted separately (F-test: 0.84–0.99 for white matter necrosis and 0.88–0.99 for vascular damage). One should also observe that the confidence intervals of the determined parameters are rather large. Fine differences in the results may be caused by small errors within the experimental dataset. Based on the above described results the following general conclusions can be drawn regarding the weak and strong points of the different models.

The sigmoid shape of dose-response for the Weibull distribution is the reverse of that of the Poisson model and may therefore be better suited for some normal tissue responses where mild damage may be more clinically relevant. On the other hand, the Lyman model is a symmetric sigmoid, which may be suboptimal for describing the response of normal tissues. The relative seriality model, having “fine-tuning” ability with the relative seriality parameter, *s* may be more suitable for fitting mixed to more serial tissues.

In the case of white matter necrosis most of the models showed low but acceptable fitting results. The Weibull distribution model was the only model giving better overall fit than the Lyman model, which was the reference model in the F-test. Furthermore, the Lyman, Parallel architecture and Weibull distribution models were best in handling the volume effect, giving the best F-test results. On the other hand, the fitting results of the inverse tumor and the critical element models were considerably worse (showing much lower Log-likelihood values) than the ones of other models, when the volume effect was taken into account, whereas when making a fit without the volume effect the Lyman model gave inferior results with the lowest Log-likelihood value.

Similarly, in the case of vascular damage, the models showed acceptable fitting results, which for many models were very good. More specifically, the Relative Seriality, Critical element, Critical volume and Parallel architecture models showed considerably better fitting results (higher Log-likelihood values) than the Inverse tumor, Lyman and Weibull distribution models when the volume dependence was taken into account. On the other hand, when the volume dependence was disregarded, apart from the Lyman and Critical volume models, the results of all the models were similar. The Critical volume, Relative Seriality, Critical element and Parallel architecture models gave the best overall fits handling volume effect in the best way, showing the lowest F-test values among all the models. On the other hand, the Lyman, Parallel architecture and Relative Seriality models gave the best fitting results (lowest F-test values) when the different spinal cord segment lengths were fitted separately.

In clinical radiotherapy there is an increasing need for accurate models capable of describing the normal tissue response as a function of the dose and the irradiated volume. The present study is an overview of the existing models that are most frequently used in scientific reports or clinical studies. However, sill more effort has to be given on radiobiological studies that could develop new improved models, which would be able to more accurately account for further biological mechanisms and will become especially suitable for biologically optimized radiotherapy.

By applying the different dose-response models on the same experimental data, their inherent structural differences could be revealed. Furthermore, the accuracy by which the volume effect is accounted for in the different models is examined. These issues have not been investigated in such a clear and systematic way before. Also, the expression of the basic dose-response parameters of the different models in terms of *D_50_* and γ_50_ is very important for their clinical implementation and it has not been reported before. It has to be stated that the size of the experimental data is rather small. However, its structure is very clear for this type of analysis and conclusions about the behavior of the different models are possible to be made. In any case, more general conclusions cannot be made because the radiobiological characteristics of the different healthy tissues vary significantly. Furthermore, the dose that is clinically delivered to these tissues is a 3-dimentional distribution, which does not provide a suitable context for demonstrating the volume effect handling by the different models.

In the literature there are several articles that deal with radiobiological studies which have evaluated and improved models based on biologically optimized clinical radiotherapy treatment plans. Although such studies may be more suitable for the extraction of clinical results in relation to a given radiobiological model, the nature of those studies is mainly to determine the values of the radiobiological model parameters regarding a given treatment technique and clinical endpoint. However, the structure of such clinical data is not suitable for performing the type of analysis that is performed in the present study in order to make a more clear inter-comparison that will reveal the inherent characteristics and differences of the examined models.

Today, all organs are assumed to be totally uniform and amorphous, whereas we know that some organ regions are more sensitive and others more tolerant to radiation. In the future, such variations need to be considered, e.g., by splitting the hilus region from the rest of the organ in most organs of mixed serial-parallel organization. Fortunately, this has not been a major problem in this study. However, if white and gray matter had been separately irradiated this would have been the case here, too.

## Figures and Tables

**Table 1.** Overview of the examined dose-response models together with a summary of their inherent parameters.

**Statistics**	**Model**	**Dose-response, *P*(*D*)**	**Dose-volume response, *P*(*D,V*)**	***Inherent model parameters***	***D*_50_ / Gy**	**γ_50_**
**Binomial**	Critial volume	(1−*e*^−*D/D*_0_^)^*N*_0_^	$\overset{\text{N}}{\underset{\text{t}=\text{M}+1}{\text{∑}}}\left(\begin{array}{l} \text{N}\\ \text{t} \end{array}\right){\text{p}}^{\text{t}}{\left(1-\text{P}\right)}^{\text{N}-\text{t}}§$	*D*_0_, *N*_0_, *M, N*	−*D*_0_ ln(1−(*M/N*)^1/*N*_0_^)	$\ln{\text{N}}_{0}{\left(1-\frac{1}{{\text{N}}_{0}}\right)}^{{\text{N}}_{0}-1}$ Implicit
	Relative seriality	${\text{e}}^{-{\text{N}}_{0}{\text{e}}^{-\left(\text{D}/{\text{D}}_{0}\right)}}$	${\left[1-{\left(1-\text{P}{\left(\text{D}\right)}^{\text{s}}\right)}^{\frac{\text{V}}{{\text{V}}_{\text{ref}}}}\right]}^{1/\text{s}}$	*D*_0_, *N*_0_, *s*	*D*_0_(ln *N*_0_−ln ln 2)	$\frac{\ln2}{2}\ln\left(\frac{{\text{N}}_{0}}{\ln2}\right)$
**Poisson**	Inverse tumor	${\text{e}}^{-N_{0}e^{-\left(\text{D}/{\text{D}}_{0}\right)}}$	${\text{e}}^{{-\text{N}}_{0}{\text{e}}^{-\left({\text{D}/\text{D}}_{0}\right)}}+\text{k}\ln\left(\text{V}/{\text{V}}_{\text{ref}}\right)$	*D*_0_, *N*_0_, *k*	*D*_0_(ln *N*_0_−ln ln 2)	$\frac{\ln2}{2}\ln\left(\frac{N_{0}}{\ln2}\right)$
	Critical element	$e^{-N_{0}e^{-\left(\text{D}/{\text{D}}_{0}\right)}}$	$1-{\left(1-\text{P}(\text{D})\right)}^{\frac{\text{V}}{{\text{V}}_{\text{ref}}}}$	*D*_0_, *N*_0_	*D*_0_(ln *N*_0_−ln ln 2)	$\frac{\ln2}{2}\ln\left(\frac{{\text{N}}_{0}}{\ln2}\right)$
**Probit**	Lyman	$\frac{\gamma_{50}}{{\text{D}}_{50}}\int_{0}^{D}e^{-\frac{1}{\pi }{\left(\gamma \frac{x-{\text{D}}_{50}}{{\text{D}}_{50}}\right)}^{2}}\text{dx}$	$\frac{1}{\sqrt{2\pi }}\int_{-\infty }^{\text{t}}e^{-\frac{t^{2}}{2}}\text{dt}\|$	*D*_50_, *m, n*	*D*_50_	$\frac{1}{\text{m}\sqrt{\pi }}$
**Logit**	Parallel architecture	$\frac{1}{1+{\left(\frac{{\text{D}}_{50}^{\text{FSU}}}{\text{D}}\right)}^{\text{k}}}$	$\frac{1}{\sqrt{2\pi \sigma^{2}}}\int_{0}^{\text{f}}{\text{e}}^{-\frac{{\text{v}-\text{v}}_{50}}{2\sigma^{2}}}\text{dv}$	*D*_50_, *k, v*_50_, *σ*	$\frac{{\text{D}}_{50}^{\text{FSU}}}{{\left(\frac{1}{{\text{v}}_{50}}-1\right)}^{1/\text{k}}}$	*k*/4
**Weibull**	Weibull distribution	$1-{\text{e}}^{-{\left(\text{D}/{\text{A}}_{1}\right)}^{{\text{A}}_{2}}}$	$1-\exp\left[-{\left({\frac{\text{D}\left(\frac{\text{V}}{{\text{V}}_{\text{ref}}}\right)}{{\text{A}}_{1}}}^{b}\right)}^{{\text{A}}_{2}}\right]$	*A*_1_, *b, A*_2_	$\frac{{\text{A}}_{1}}{{\left(\ln2\right)}^{1/{\text{A}}_{2}}}$	$\frac{{\text{A}}_{2}\ln2}{2}$
Binomial	Critial volume	(1−*e*^−D/D_0_^)^*N*_0_^	$\sum_{\text{t}=\text{M}+1}^{\text{N}}\left(\begin{array}{l} \text{N}\\ \text{t} \end{array}\right){\text{P}}^{\text{t}}{\left(1-\text{P}\right)}^{\text{N}-\text{t}}§$	D0, N0, M, N	−*D*_0_ ln(1−(*M/N*)^1/N_0_^)	$\ln{\text{N}}_{0}{\left(1\text{−}\frac{1}{{\text{N}}_{0}}\right)}^{{\text{N}}_{0}-1}$ Implicit
	Relative seriality	$e^{-{\text{N}}_{0}e^{-\left(\text{D}/{\text{D}}_{0}\right)}}$	${\left[1-{\left(1-\text{P}{\left(\text{D}\right)}^{\text{s}}\right)}^{\frac{\text{V}}{{\text{V}}_{\text{ref}}}}\right]}^{1/\text{s}}$	D0, N0, s	*D*_0_(ln *N*_0_−ln ln 2)	$\frac{\ln2}{2}\ln\left(\frac{{\text{N}}_{0}}{\ln2}\right)$
Poisson	Inverse tumor	$e^{-{\text{N}}_{0}e^{-\left(\text{D}/{\text{D}}_{0}\right)}}$	${\text{e}}^{-{\text{N}}_{0}e^{-\left(\text{D}/{\text{D}}_{0}\right)}}+\text{K ln}\left(\text{V}/{\text{V}}_{\text{ref}}\right)$	D0, N0, k	*D*_0_(ln *N*_0_−ln ln 2)	$\frac{\ln2}{2}\ln\left(\frac{{\text{N}}_{0}}{\ln2}\right)$
	Critical element	$e^{-{\text{N}}_{0}e^{-\left(\text{D}/{\text{D}}_{0}\right)}}$	$1-{\left(1-\text{P}(\text{D})\right)}^{\frac{\text{V}}{{\text{V}}_{\text{ref}}}}$	D0, N0	*D*_0_(ln *N*_0_−ln ln 2)	$\frac{\ln2}{2}\ln\left(\frac{{\text{N}}_{0}}{\ln2}\right)$
**Probit**	Lyman	$\frac{\gamma_{50}}{{\text{D}}_{50}}\int_{0}^{\text{D}}{\text{e}}^{\frac{1}{\pi }{\left(\gamma \frac{x-{\text{D}}_{50}}{{\text{D}}_{50}}\right)}^{2}}\text{dx}$	${\frac{1}{\sqrt{2\pi }}\int_{-\infty }^{\text{t}}{\text{e}}^{-\frac{{\text{t}}^{2}}{2}}\text{dt}}_{\|}$	D50, m, n	*D*_50_	$\frac{1}{\text{m}\sqrt{\pi }}$
**Logit**	Parallel architecture	$\frac{1}{1+{\left(\frac{{\text{D}}_{50}^{\text{FSU}}}{\text{D}}\right)}^{\text{k}}}$	$\frac{1}{\sqrt{2\pi \sigma^{2}}}\int_{0}^{\text{f}}{\text{e}}^{-\frac{\text{v}-{\text{v}}_{50}}{2\sigma^{2}}}\text{dv}$	D50, k, v50, σ	$\frac{{\text{D}}_{50}^{\text{FSU}}}{{\left(\frac{1}{{\text{v}}_{50}}-1\right)}^{1/\text{k}}}$	k/4
**Weibull**	Weibull distribution	$1-{\text{e}}^{-{\left(\text{D}/{\text{A}}_{1}\right)}^{{\text{A}}_{2}}}$	$1-\exp\left[-{\left(\frac{\text{D}{\left(\frac{\text{V}}{{\text{V}}_{\text{ref}}}\right)}^{\text{b}}}{{\text{A}}_{1}}\right)}^{{\text{A}}_{2}}\right]$	A1, b, A2	$\frac{{\text{A}}_{1}}{{\left(\ln2\right)}^{1/{\text{A}}_{2}}}$	$\frac{{\text{A}}_{2}\ln2}{2}$

$§P=P(D)\left(\frac{V}{V_{\text{ref}}}\right)$; ‖ *t* is given by equation (19)

**Table 2.** Dose-response data for developing white matter related spinal cord paralysis (white matter necrosis) within 30 weeks and paralysis or histological evidence of vascular lesions (vascular damage) after a latent interval of >30 weeks after single dose irradiation of the rat spinal cord [24].

**Endpoint**	**Field Length (mm)**	**Dose (Gy)**	**Responders (#)**	**Group size (#)**
**White matter necrosis**	16	20	0	6
		21	3	6
		22	3	6
		23	6	6

	8	22	0	6
		24	1	6
		28.5	2	6
		32.5	4	6
		40	5	5

	4	39.1	0	6
		42.7	1	6
		47.8	2	6
		54.5	4	6
		70	6	6

**Vascular damage**	16	18	0	6
		20	3	6
		21	3	3

	8	20.4	1	6
		22	4	6
		24	4	5
		28.5	4	4

	4	25.3	4	6
		30	4	6
		35.9	5	6
		39.1	6	6

**Table 3.** Model parameter values for white matter necrosis. The best estimates of the parameter values are given with their 68% confidence intervals. The values of the Log-likelihood function, χ^2^, degrees-of-freedom (DF) and probability of χ^2^ distribution (*P*_χ_) that describe the goodness-of-fit of the models to the experimental data, with and without accounting for the volume effect. The inter-comparison of the models is performed, the AIC measure and the F-test, using the Lyman model as reference (with the volume effect).

**Name of the model**	***D*_50_ (Gy)**	**γ50**	**Volume parameters**	**Volume effect**	**Log likelihood**	**χ^2^**	**DF**	***P*_χ_**	**AIC**	***F-test***
Critical volume	20.7 (20.3–21.2)	3.20 (2.6–3.3)	*M* = 20 (18–21) *N* = 397 (320–431)	With	−35.6	12.75	10	0.24	20.75	0.57
				Without	−32.0	4.57	8	0.80	16.57	0.92
Relative seriality	21.3 (21.1–21.5)	4.0 (3.6–4.4)	*s* = 0.01 (0.01–0.01)	With	−35.3	14.09	11	0.23	20.09	0.63
				Without	−31.9	4.84	8	0.77	16.84	0.93
Inverse tumor	19.5 (17.5–21.5)	1.0 (0.8–1.2)	*k* = −3.1 (−3.6–−2.5)	With	−40.2	16.89	11	0.11	22.89	0.73
				Without	−31.9	4.84	8	0.77	16.84	0.96
Critical element	24.2 (23.1–26.0)	1.0 (0.7–1.3)	*s* = 1.0	With	−47.3	25.76	12	0.01	29.76	0.90
				Without	−31.9	4.84	8	0.77	16.84	0.99
**Lyman (Gaussian) Reference model**	**21.0 (20.5**–**21.7)**	**4.0 (3.3**–**4.7)**	***n* = 0.62 (0.58–0.66)**	**With**	**−36.0**	**11.46**	**11**	**0.41**	**17.46**	**0.50**
				Without	−33.4	5.56	8	0.70	17.56	0.84
Parallel architecture	21.2 (18.0–30.4)	3.9 (3.2–5.1)	*v*_50_ = 0.14 (0.13–0.16) *σ_v_* = 0.03 (0.02–0.03)	With	−34.3	11.52	10	0.32	19.52	0.51
				Without	−32.1	5.02	8	0.76	17.02	0.87
Weibull distribution	22.8 (22.6–23.4)	3.2 (2.5–4.0)	*b* = 0.63 (0.59–0.66)	With	−35.4	10.33	11	0.50	16.33	0.43
				Without	−31.6	4.26	8	0.83	16.26	0.89

**Table 4.** Model parameter values for vascular damage.

**Name of the model**	***D*_50_ (Gy)**	**γ50**	**Volume parameters**	**Volume effect**	**Log likelihood**	**χ^2^**	**DF**	***P*_χ_**	**AIC**	***F-test***
Critical volume	19.3 (19.0–19.7)	5.3 (4.1–6.2)	*M* = 1 (0–10) *N* = 14 (6–40)	With	−26.2	4.11	7	0.77	12.11	0.08
				Without	−25.1	2.21	5	0.82	14.21	0.93
Relative seriality	19.9 (19.4–20.4)	6.7 (5.5–8.0)	*s* = 0.28 (0.15–0.53)	With	−26.1	5.03	8	0.75	11.03	0.11
				Without	−24.9	1.51	5	0.91	13.51	0.90
Inverse tumor	20.2 (19.0–20.8)	2.0 (1.5–2.5)	*k* = −0.9 (−1.4–−0.4)	With	−30.6	13.17	8	0.11	19.17	0.53
				Without	−24.9	1.51	5	0.91	13.51	0.99
Critical element	20.2 (19.7–20.3)	4.9 (3.9–6.1)	*s* = 1.0	With	−26.8	5.62	9	0.78	9.62	0.13
				Without	−24.9	1.51	5	0.91	13.51	0.92
**Lyman (Gaussian) Reference model**	**19.6 (18.4**–**20.4)**	**2.6 (1.8**–**3.6)**	***n* = 0.20 (0.13**–**0.27)**	**With**	**−30.9**	**12.41**	**8**	**0.13**	**18.41**	**0.50**
				Without	−26.7	4.19	5	0.52	16.19	0.88
Parallel architecture	19.9 (19.0–21.5)	7.9 (5.9–8.2)	*v*_50_ = 0.17 (0.14–0.19) *σ_v_* = 0.07 (0.06–0.09)	With	−26.7	5.50	7	0.60	13.5	0.15
				Without	−24.7	1.80	5	0.88	13.8	0.88
Weibull distribution	22.9 (21.9–23.5)	1.6 (1.2–2.0)	*b* = 0.20 (0.13–0.27)	With	−31.1	12.20	8	0.14	18.2	0.49
				Without	−24.7	1.97	5	0.85	13.97	0.97
